# Prior to extension, Transcriptomes of fibroblast-like Synoviocytes from extended and Polyarticular juvenile idiopathic arthritis are indistinguishable

**DOI:** 10.1186/s12969-017-0217-6

**Published:** 2018-01-08

**Authors:** AnneMarie C. Brescia, Megan M. Simonds, Suzanne M. McCahan, Kathleen E. Sullivan, Carlos D. Rose

**Affiliations:** 1 Pediatric Rheumatology, Nemours/AI DuPont Hospital for Children, 1600 Rockland Road, Wilmington, DE 19803 USA; 2 Nemours Biomedical Research, 1600 Rockland Road, Wilmington, DE USA; 3 Pediatric Immunology, Children’s Hospital of Philadelphia, 3615 Civic Center Boulevard, Philadelphia, PA USA

**Keywords:** Juvenile idiopathic arthritis, Microarray., Gene expression., Transcriptome., Extended JIA., JIA subtypes., Fibroblast-like synoviocytes., Biomarkers.

## Abstract

**Background:**

Our intent was to identify differences between the transcriptome of fibroblast-like synoviocytes (FLS) in oligoarticular juvenile idiopathic arthritis (JIA) before extension when compared to persistent subtype of JIA, when the two are clinically indistinguishable. Additionally, we sought to determine if differences between the transcriptomes of FLS from extended-to-be and polyarticular course JIA could be detected. Our hypothesis was that intrinsic differences in the transcriptome of the FLS from extended-to-be JIA would distinguish them from persistent oligoarticular JIA, before the course is clinically apparent.

**Methods:**

Global gene expression was defined in cultured FLS from 6 controls, 12 JIA with persistent course, 7 JIA prior to extension (extended-to-be), 4 JIA with extended course and 6 polyarticular onset, using Affymetrix Human GeneChips 133plus2.0.

**Results:**

Bioconductor Linear Models for Microarray Analysis revealed 22 probesets with differential expression between persistent and extended-to-be FLS at 15% FDR, however only 2 probesets distinguished extended-to-be from extended and none distinguished extended-to-be and polyarticular at 15% FDR. Differences in extended and polyarticular gene expression profiles were not detected. Confirmation of select genes was done on the RNA level by RT-qPCR and on the protein level in synovial fluid by ELISA.

**Conclusions:**

The transcriptome of FLS from extended-to-be juvenile idiopathic arthritis is distinct from persistent course before a clinical distinction can be made. Additionally, the transcriptome of extended-to-be and polyarticular course, including those who have already extended, are indistinguishable. These gene expression data suggest that FLS already reflect a polyarticular behavior early in disease course, suggesting that extended-to-be may be “latent polyarticular” at onset. These differences can be used to develop early biomarkers of disease course, allowing for better-informed treatment decisions.

**Electronic supplementary material:**

The online version of this article (10.1186/s12969-017-0217-6) contains supplementary material, which is available to authorized users.

## Background

Juvenile Idiopathic Arthritis (JIA), the most common rheumatic disease of childhood [1], carries risk of permanent disability. Oligoarticular subtype is the most benign, except when it evolves into polyarticular course, termed extended oligoarticular disease. Persistent oligoarthritis affects up to 4 joints, while extended oligoarthritis affects a cumulative total of 5 or more joints after the first 6 months. Between 21 and 50% of patients show extension to polyarticular course, of whom only 13–23% achieve remission [2]. Functional outcome for children with extended oligoarticular JIA can be poor and the key to achieving improvement may be in prediction of extension so that early treatment can be initiated [3].

In rheumatoid arthritis (RA), fibroblast-like synoviocytes (FLS) are cytokine producing key effector cells that both directly destroy cartilage and promote perpetuation of inflammation [4]. These cells have a central role in the pathogenesis of rheumatoid synovitis and may provide insight into differences in the pathogenic processes that allow extension to polyarticular course in JIA. We hypothesize that FLS contain central signals involved in the “switch” from oligoarticular to polyarticular course.

This is the first study to look at the comparative transcriptomes of JIA FLS of different disease subtypes. Transcriptome analysis revealed important differences in gene expression of FLS from oligoarticular JIA prior to extension (extended-to-be) when compared to that of FLS from persistent oligoarticular subtype. Moreover, no differences were detected in the transcriptomes of FLS from polyarticular courses (extended and polyarticular subtypes) and FLS of extended-to-be JIA, early in disease course. Differences in JIA FLS transcriptomes between persistent and extended-to-be challenge the classification criteria and can provide clues for candidate biomarker development to predict disease course in JIA.

## Methods

### Patients and samples

As part of an ongoing Institutional Review Board (IRB) -approved protocol, remnant synovial fluids/tissues were obtained from arthrocenteses and synovectomies from five groups: [1] controls (C), [2] persistent oligoarticular (PR), [3] oligoarticular prior to extension (Ext-to-be), [4] oligoarticular with extended course (E), and [5] polyarticular onset (Poly). Medical record review was conducted to select appropriate samples from our synovial fluid and tissue repository. All patients met the ILAR classification criteria for JIA [5], including persistent and extended oligoarticular and polyarticular subtypes, specifically excluding enthesitis-related and psoriatic subtypes. Clinical information on individual patients is in supplemental table (Additional file 1 Clinical Table). There were 6 C, 12 PR, 7 Ext-to-be, 4 E and 6 Poly included in the study. All control samples were obtained from orthopedic procedures. Patients with persistent oligoarticular course were followed for between 2 and 18 years from diagnosis without extension. The majority of patients were antinuclear antibody (ANA) positive. All patients who had documented testing for HLA-B27 allele and cyclic citrullinated peptide (CCP) antibody were negative. Of those tested, one patient with extended oligoarticular course was rheumatoid factor (RF) positive.

### Cell culture

Synovial fluid/tissue was plated in 6-well plates with 15% fetal bovine serum/Dulbecco’s modified Eagle’s medium to establish primary cultures of FLS, passaged 3–6 times, then harvested at confluence. By the third passage, FLS are the predominant cell type in culture [6]. As indicated in the supplemental table, four of the control FLS were grown from synovial tissue and the remainder of the FLS were grown from synovial fluid.

### Gene expression

RNA was extracted from cultured FLS using TRIzol, purified using Qiagen RNeasy Mini Prep kit, amplified, fluorescence labeled and hybridized to GeneChip Human Genome U133 Plus 2.0 Arrays per Affymetrix protocol. Arrays were stained using GeneChip Hybridization, Wash, and Stain kit (Affymetrix), and scanned and analyzed using Affymetrix command and expression console software. Log2 expression values were calculated for the 54,675 probesets with GeneChip Robust Multiarray Analysis (GC-RMA), a method of microarray normalization and summarization, using the gcRMA Bioconductor package [7].

### Gene expression analysis

Data was filtered for log2 expression >4 in all hybridizations in at least one group in each pairwise comparison, then for |1.5|-fold change. Probesets with a statistically significant difference in expression in pairwise comparisons were identified using Bioconductor package Linear Models for Microarray Analysis (LIMMA) with Benjamini and Hochberg’s method to control the false-discovery rate (FDR) [8], with significance of 15%. Hierarchical clustering was performed using Euclidean correlation and average linkage in MeV (MultiExperiment Viewer) [9].

### Quantitative reverse transcription-polymerase chain reaction (qRT-PCR)

Agilent Bioanalzer and Nanodrop 2000 were used for quantification of RNA. High-Capacity cDNA RT Kit from LifeTechnologies was used (manufacturer’s procedures) with ABI Veriti thermal cycler. After reverse transcription, 20ul nuclease-free water was added (total volume 40ul). cDNA was assayed on 7900HT Real-Time PCR system with TaqMan Gene Expression Assays. TaqMan IDs for each gene are as follows: CLDN11 Hs00194440_m1, ANKRD44 Hs00403517_m1, ICAM2 Hs00609563_m1, KLHL13 Hs01006506_m1, LY6K Hs03988347_m1, KIF11 Hs00189698_m1, MAMLD1 Hs00193976_m1, RAB27B Hs00188156_m1, RGS2 Hs01009070_g1, ZNF204P Hs00394812_m1. Samples (in triplicate) on 384 well plate were cycled on 7900HT Real-Time PCR system following manufacturer’s default conditions using SDS software v2.4 relative quantification run. Data was analyzed using the RQ (relative quantification) Manager to calculate relative quantification using the delta-delta-Ct (ddCt) method [10].

### Enzyme-linked immunosorbent assay (ELISA)

Using synovial fluid samples from 12 PR and 7 Ext-to-be, CD14 ELISA was completed using Quantikine Colormetric ELISA kits from R&D Systems (manufacturer’s protocol). For the other proteins, we performed standard sandwich ELISAs optimized by our lab [11]. Reagents: MBP (Novus NB110-79873B, NBP2–46632), KLHL13 (SCBT 138378, bs-7752R), HspBAP1 (Novus NBP1–92014, LifeSpan LS-C371907–50), ANKRD44 (Novus NBP1–80887, LifeSpan LS-C251405–200). Averages were obtained using Excel. Standard errors and *p*-values were calculated using SigmaStat.

## Results

The transcriptome of FLS from extended-to-be JIA was distinct from the transcriptome of FLS of persistent oligoarticular JIA. We sought to identify potential candidate biomarkers for polyarticular course through differences in FLS gene expression. Gene expression profiling was done of cultured FLS from 6 controls (C), 12 persistent oligoarticular (PR), 7 extended-to-be, obtained prior to extension (Ext-to-be), 4 extended, obtained after disease had extended (E) and 6 polyarticular, with ≥5 joints involved in first 6 months of disease (Poly) samples. A large number of differences between controls and the JIA groups were identified. (Additional file 2 FDR_results_table).

**Fig. 1:**
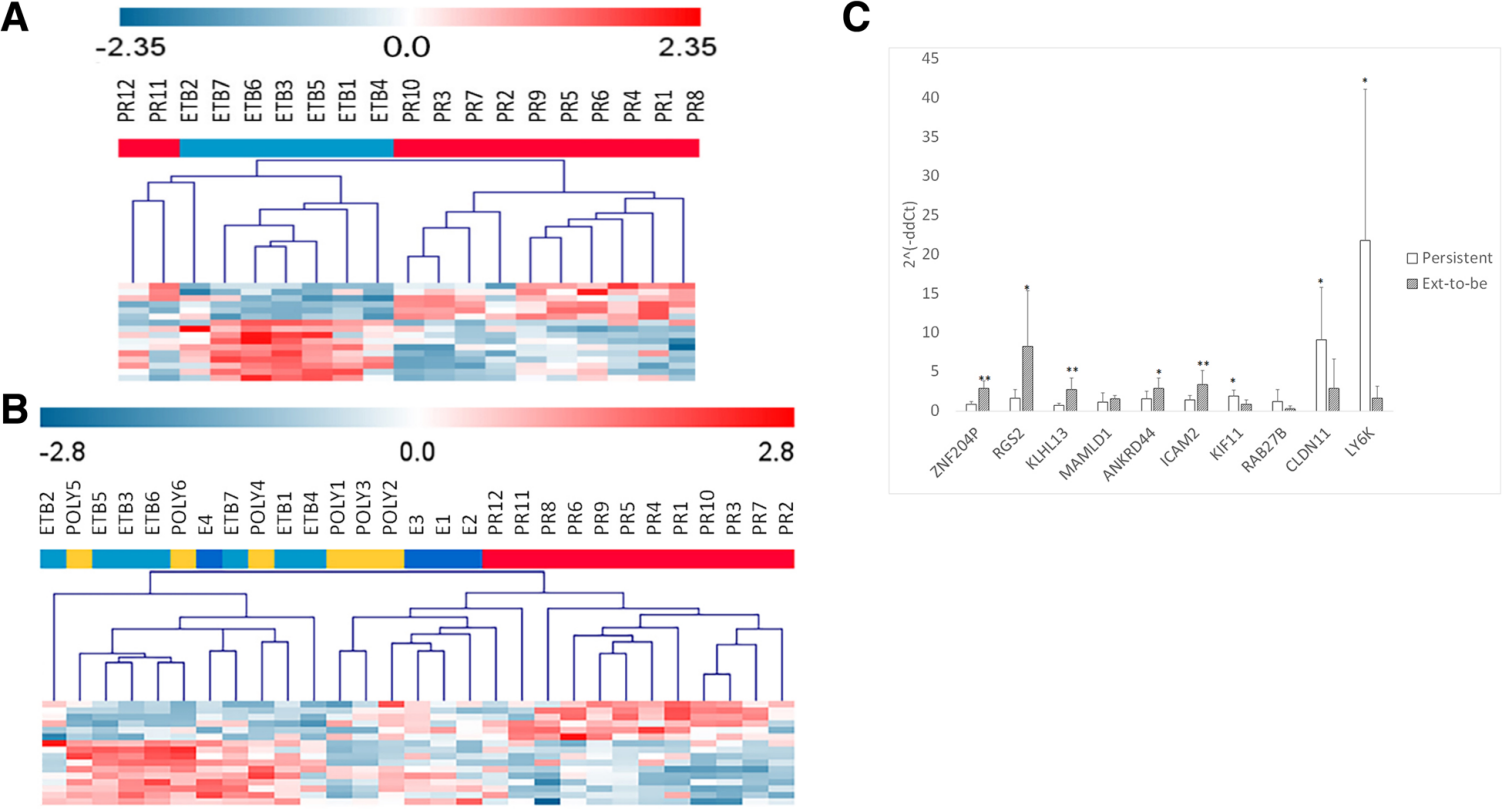
Differentially expressed genes and confirmation on RNA level **a**. Hierarchal clustering of 16 differentially expressed genes between persistent oligoarticular JIA (PR, red) and extended-to-be samples (ETB, light blue) separates 10 of the 12 persistent from extended-to-be, clusters the extended-to-be together and separates extended-to-be from most of the persistent. **b**. Hierarchal clustering of the same 16 genes based on the expression patterns in PR (red), ETB (light blue), extended (E, dark blue), and polyarticular (poly, yellow). The PR samples cluster separately from the other three groups, which cluster together. **c**. qRT-PCR confirmation of 10 select genes. * p-value <0.05; ** *p*-value <0.01. MAMLD1 and RAB27B did not meet statistical significance for differential expression by PCR when comparing FLS RNA from persistent and extended—to-be (Ext-to-be) JIA

We directed our studies to define differences in the transcriptome of persistent when compared to extended-to-be JIA, prior to extension, when the two subtypes are clinically indistinguishable. Of the probesets with log2 expression >4 for all hybridizations in PR or Ext-to-be, 2856 probesets had ≥ |1.5|-fold change when comparing 12 PR and 7 Ext-to-be samples. LIMMA analysis of these 2856 probesets identified 22 differentially expressed probesets at 15% FDR. XIST appeared 6 times in this group and was removed to minimize bias from clustering, since all 7 of the Ext-to-be samples were from females while 7/12 PR samples were from females**.** Hierarchical clustering of the 16 differentially expressed probesets (Table 1) demonstrated excellent separation of the two groups, with 2 exceptions (Fig. 1a).

**Table 1:** Differentially Expressed Genes Between Persistent and Extended-to-be Oligoarticular JIA at 15% False Discovery Rate

Symbol	Gene Title	Fold Change	Upregulated in
ZNF204P	Zinc finger protein 204, pseudogene	6.00	Extended-to-be
RGS2	Regulator of G-protein signaling 2	7.49	Extended-to-be
KLHL13	Kelch-like family member 13	3.90	Extended-to-be
GPRASP1	G protein-couple receptor associated sorting protein 1	4.59	Extended-to-be
SPINK13	Serine peptidase inhibitor, Kazal type 13 (putative)	14.50	Extended-to-be
MAMLD1	Mastermind-like domain containing 1	2.55	Extended-to-be
ANKRD44	Ankyrin repeat domain 44	2.97	Extended-to-be
ICAM2	Intercellular adhesion molecule 2	2.85	Extended-to-be
ABCC6	ATP binding cassette subfamily C member 6	2.93	Extended-to-be
HLX	H2.0-like homeobox	2.62	Extended-to-be
KIF11	Kinesin family member 11	6.50	Persistent
RAB27B	RAB27B, member RAS oncogene family	6.53	Persistent
TOP2A	Topoisomerase (DNA) II alpha	14.49	Persistent
CLDN11	Claudin 11	4.49	Persistent
LY6K	Lymphocyte antigen 6 complex, locus K	7.27	Persistent
GEN1	GEN1 Holliday junction 5′ flap endonuclease	2.21	Persistent

Six probesets were more highly expressed in the persistent and 10 probesets more highly expressed in the extended-to-be FLS. RNA level confirmation by qRT-PCR was done for a representative group of 10 genes from the 16 differentially expressed genes (Fig. 1c). The real-time PCR confirmation was done on RNA from the same samples as used for the microarray analysis. The PCR results were consistent with the microarray finding for 8 of the 10 tested genes, with the exceptions of MAMLD1 and RAB27B.

### No differences were detected between the transcriptome of FLS of extended-to-be oligoarticular JIA and that of FLS from polyarticular course JIA

Once we demonstrated that gene expression of FLS from Ext-to-be was distinct from that of FLS from persistent course, we sought to determine if FLS from Ext-to-be were distinguishable from those of polyarticular course. In a pairwise comparison of extended-to-be and extended samples, there were only 2 significant differentially expressed probesets by 15% FDR. Pairwise comparison of polyarticular versus either Ext-to-be or E revealed no differentially expressed probesets (lowest adjusted *p*-values 0.42 and 0.33, respectively).

### Expression pattern of differentially expressed genes in FLS from polyarticular and extended JIA

Since there were no detectible differences between gene expression of Ext-to-be and polyarticular course, we turned our attention to the expression patterns of the 16 differentially expressed genes between PR and Ext-to-be. Patterns of expression of these genes in the E and Poly samples demonstrated close correlation with the pattern seen in the Ext-to-be when compared with PR (Fig. 1b).

### Secreted proteins as candidate synovial fluid biomarkers for prediction of course in JIA

**Fig. 2:**
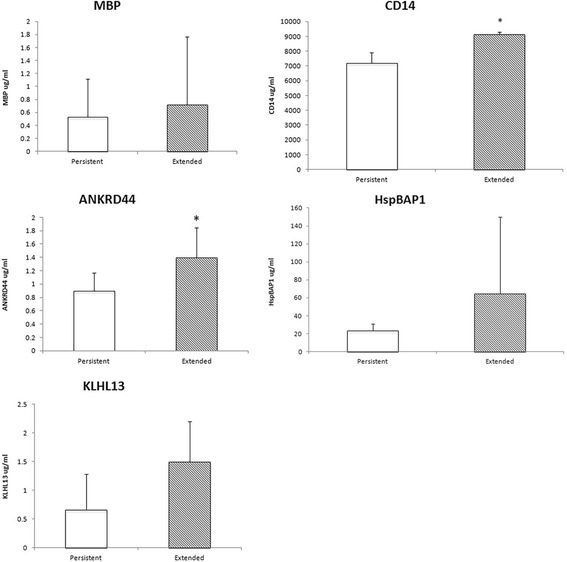
ELISA confirmation of secreted proteins on synovial fluid samples, from the same patients used in the transcriptome analysis. Synovial fluid from all 12 persistent and the combined group of all 11 extended and extended-to-be samples were assayed for levels of specific proteins. All 5 genes were more highly expressed by microarray in FLS of extended-to-be. All 5 proteins trended towards increased expression in the synovial fluid of extended and extended-to-be, but only CD14 and ANKRD44 differences reached significance at *p*-value <0.05

To translate differences in FLS gene expression into a clinically accessible rapid through-put assay, we tested synovial fluid by ELISA for secreted proteins. Starting with the 16 differentially expressed proteins (Table 1) we selected two genes, ANKRD44 and KLHL13, which encode secreted proteins, Ankyrin and Kelch respectively, for testing by ELISA on acellular synovial fluid. In order to expand our pool of potential biomarkers, we selected 3 additional genes from the pool of probesets with adjusted *p*-values < 0.20. The secreted proteins from these genes are CD14, myelin basic protein (encoded by MBP) and HSP70-binding protein (encoded by HspBAP1). ELISA results for levels of these proteins are shown in Fig. 2. Differences in protein levels of Ankyrin and CD14 in the synovial fluid of PR versus ext-to-be reached statistical significance.

## Discussion

Our hypothesis was that intrinsic differences in the transcriptome of the FLS from extended-to-be JIA early in the course would distinguish them from persistent oligoarticular JIA, before the course is clinically apparent.

We performed gene expression profiling using FLS from controls and patients with differing disease courses, specifically persistent oligoarticular, extended-to-be and polyarticular course (extended and polyarticular onset). The extended-to-be samples were all taken from the very first sample available, which preceded extension in all patients. For the 16 differentially expressed genes, Ext-to-be already display expression patterns similar to the FLS of patients with polyarticular course. This pattern of gene expression is distinct from oligoarticular who don’t extend (persistent), demonstrating that there are biologic differences prior to detectible clinical differences between PR and Ext-to-be. Taken together, these data indicate a specific pattern of gene expression in FLS of polyarticular course (Poly and E) that is displayed by FLS of Ext-to-be even before that course is clinically apparent.

Ext-to-be FLS demonstrated increased expression of HLX, a homeobox transcription factor, which is a positive regulator of Th1 cell differentiation and a negative regulator of Th2 cell differentiation. Conversion of Th17 cells to non-classic Th1 phenotype has been demonstrated in the inflamed joint in JIA [12] and FLS may influence this altered T-cell repertoire. ICAM-2 (CD102) is an intercellular adhesion molecule that binds LFA-1 protein. It mediates adhesion and has a role in antigen-specific immune response, NK-cell mediated clearance and lymphocyte recirculation. Singh et al. have proposed ICAM-2 as a potential therapeutic target to inhibit FLS activation in RA as they demonstrated that T-cell induced activation of Akt in FLS is mediated by ICAM-2 [13]. Unexpectedly, CD14 was increased in Ext-to-be FLS on the RNA level, which translated into increased protein in the synovial fluid. A novel procollagen + CD45 + CD14 + IL17RA + CD34- fibrocyte-like cell (FLC) has been reported in the synovial fluid of JIA and the authors postulated an FLC-CD8 T cell role in perpetuation of inflammation in JIA [14]. In RA, increased soluble CD14 (sCD14) has been described in plasma and synovial fluid, with thoughts that sCD14 was produced by RA synovial macrophages through cleavage of membranous CD14. These authors postulated that RA synovial fibroblasts were sensitive to LPS in the presence of sCD14 and LPS-binding protein [15]. One could argue that increased monocytes in Ext-to-be synovial fluid could increase CD14 level, however, the proportion of monocytes/macrophages in the synovial fluid mononuclear cells was not significantly different between PR and Ext-to-be [16], pointing towards the FLS as important contributors to differences in CD14 levels in the synovial fluid.

There are small numbers of samples and we may be underpowered to detect differences between certain groups, however, we did identify differences between the transcriptomes of PR and Ext-to-be FLS. This is the first study looking at direct patient samples of JIA FLS in this way. Even with small sample numbers in the comparisons, there were large numbers of differentially expressed genes between controls and each of the other groups. We were very stringent in our selection of samples to ensure that samples used met the ILAR criteria and extended-to-be were obtained prior to extension. In 8 of 10 genes tested by qRT-PCR, we were able to confirm the microarray findings. We removed XIST to remove some bias due to sex differences. Samples PR11 and PR12 cluster more closely with the Ext-to-be samples. PR11 was from a patient on naproxen who was followed for 9 years without extension. PR12 was from a patient who remained persistent in course (7 years follow up), however, was already on methotrexate. Ultimately, this patient required advanced therapy with anti-TNF agent for refractory uveitis, raising the question of altered course of arthritis through biologic therapy.

Although significant work has been done in FLS from RA, there is minimal comparable work done in JIA in general and none in the FLS from JIA. This is the first study to compare global gene expression of FLS from persistent, extended-to-be and polyarticular course JIA. Through this transcriptome level comparison, this study demonstrates that prior to extension, the FLS of JIA show a gene expression pattern that is more closely aligned to that of polyarticular course JIA than to that of persistent oligoarticular JIA. Using this information, we can develop prognostic synovial fluid biomarkers for predicting extension of oligoarticular JIA to polyarticular course, thereby allowing for more informed treatment decisions earlier in the course, perhaps improving outcomes for children with arthritis.

## Conclusions

This correlation of early gene expression in the extended-to-be FLS with that of already extended and polyarticular FLS highlights the importance of FLS in reflecting ultimate course of disease. Rather than being persistent JIA that later evolves into an extended pattern, these data are the first pieces of evidence to suggest that extended-to-be patients may have “latent polyarticular” arthritis and perhaps should be treated more aggressively earlier in disease course. The FLS may hold the key for determining whether there is a pre-programmed prognosis or whether extended-to-be JIA already has smoldering polyarticular involvement that is below the limit of clinical detection. Our future goal is to develop a rapid throughput synovial fluid biomarker panel for predicting course in oligoarticular JIA. Demonstrating the translation of increased RNA expression into increased protein levels allows us to move forward with screening of potential synovial fluid biomarkers.

## Additional files

Additional file 1: Clinical Table (XLSX 41 kb)

Additional file 2: FDR_results_table (XLSX 11 kb)
